# When local impedance meets contact force: preliminary experience from the CHARISMA registry

**DOI:** 10.1007/s10840-022-01163-7

**Published:** 2022-03-24

**Authors:** Francesco Solimene, Valerio De Sanctis, Ruggero Maggio, Maurizio Malacrida, Luca Segreti, Matteo Anselmino, Vincenzo Schillaci, Massimo Mantica, Marco Scaglione, Antonio Dello Russo, Filippo Maria Cauti, Gianluca Zingarini, Claudio Pandozi, Marco Cavaiani, Anna Ferraro, Giampiero Maglia, Giuseppe Stabile

**Affiliations:** 1 Clinica Montevergine, Avellino, Mercogliano Italy; 2 Department of Cardiac Electrophysiology and Pacing, Istituto Clinico Sant’Ambrogio, Via Luigi Giuseppe Faravelli, 16, 20149 Milan, Italy; 3 Infermi Hospital, Rivoli, Italy; 4 Boston Scientific, Milan, Italy; 5 Second Division of Cardiology, Cardiac-Thoracic-Vascular Department, New Santa Chiara Hospital, Azienda Ospedaliero Universitaria Pisana, Pisa, Italy; 6 Division of Cardiology, “Città della Salute e della Scienza di Torino” Hospital, Department of Medical Sciences, University of Turin, Turin, Italy; 7 Cardinal Massaia Hospital, Asti, Italy; 8 Cardiology and Arrhythmology Clinic, Marche Polytechnic University, Ancona, Italy; 9 Arrhythmology Unit, Ospedale San Giovanni Calibita, Fatebefratelli, Isola Tiberina, Rome, Italy; 10 Ospedale Santa Maria della Misericordia, Perugia, Italy; 11 Division of Cardiology, San Filippo Neri Hospital, Rome, Italy; 12 Azienda Ospedaliera Pugliese Ciaccio, Catanzaro, Italy; 13 Anthea Hospital, Bari, Italy

**Keywords:** Atrial fibrillation, Catheter ablation, Local impedance, Contact force, Lesion formation, PVI

## Abstract

**Purpose:**

Highly localized impedance (LI) measurements during atrial fibrillation (AF) ablation have emerged as a viable real-time indicator of tissue characteristics and the consequent durability of the lesions created. We investigated the impact of catheter-tissue contact force (CF) on LI behavior during pulmonary vein isolation (PVI).

**Methods:**

Forty-five consecutive patients of the CHARISMA registry undergoing *de novo* AF radiofrequency (RF) catheter ablation with a novel open-irrigated-tip catheter endowed with CF and LI measurement capabilities (Stablepoint™ catheter, Boston Scientific) were included.

**Results:**

A total of 2895 point-by-point RF applications were analyzed (RF delivery time (DT) = 8.7±4s, CF = 13 ±±8 g, LI drop = 23 ±±7 Ω). All PVs were successfully isolated in an overall procedure time of 118 ±±34 min (fluoroscopy time = 13 ±±8 min). The magnitude of LI drop weakly correlated with CF (*r* = 0.13, 95% confidence interval (CI): 0.09 to 0.16, *p* < 0.0001), whereas both CF and LI drop inversely correlated with DT (*r* = −0.26, 95%CI: −0.29 to −0.22, *p* < 0.0001 for CF; *r* = −0.36, 95%CI: −0.39 to −0.33, *p* < 0.0001 for LI). For each 10 g of CF, LI drop markedly increased from 22.4 ± 7 Ω to 24.0 ± 8 Ω at 5 to 25 g CF intervals (5–14 g of CF vs 15–24 g of CF, *p* < 0.0001), whereas it showed smooth transition over 25 g (24.8 ± 7Ω at ≥ 25 g CF intervals, *p* = 0.0606 vs 15–24 g of CF). No major complications occurred during the procedures or within 30 days.

**Conclusions:**

CF significantly affects LI drop and probable consequent lesion formation during RF PVI. The benefit of higher contact (> 25 g) between the catheter and the tissue appears to have less impact on LI drop.

**Trial registration:**

Catheter Ablation of Arrhythmias With High Density Mapping System in the Real World Practice (CHARISMA). URL: http://clinicaltrials.gov/ Identifier: NCT03793998

**Supplementary Information:**

The online version contains supplementary material available at 10.1007/s10840-022-01163-7.

## Introduction

Catheter ablation aimed at pulmonary vein (PV) isolation is the most effective treatment in patients with atrial fibrillation (AF) and is now recommended as the first-line therapy [1, 2]. Despite acute safety and efficacy, a considerable number of recurrences are observed during long-term follow-up, mainly as a result of PV reconnection [1–3]. Several strategies have been proposed in order to achieve durable, transmural lesions, thereby improving the efficiency of catheter ablation [4]. Highly localized impedance (LI) measurements during AF ablation have emerged as a viable real-time indicator of tissue characteristics and the consequent durability of the lesions created [5–7]. A recently released catheter has combined CF detection with LI assessment in a single catheter-tissue contact force (CF)-LI catheter [8]. In swine and *in vitro*, the addition of LI to CF has provided feedback on both electrical and mechanical loads and allows the evaluation of tissue resistivity, and thus of the type of tissue with which the catheter is in contact. It has also provided feedback on whether volumetric tissue heating is inadequate, sufficient, or excessive. In addition, in a point-by-point workflow with consistent CF, the visualization of LI significantly reduced RF time [8]. We investigated the impact of CF on LI behavior during PV isolation.

## Methods

### Patient population and study design

CHARISMA was a prospective, multi-center cohort study designed to describe Italian clinical practice regarding the approach to ablation of various arrhythmias. The study complied with the Declaration of Helsinki, the locally appointed ethics committee approved the research protocol, and informed consent was obtained from all patients. From January 2021 to July 2021, 45 consecutive patients indicated for AF ablation who were undergoing their first high-resolution mapping and ablation procedure with a novel CF- and LI-featured catheter in 9 Italian centers were included in our analysis. All patients were followed up at the same hospital, from the time of first ablation to the last follow-up visit.

### Ablation procedure

After completion of the baseline evaluation, patients underwent ablation in accordance with standard clinical practice guidelines [1]. All procedures were performed under conscious sedation or general anesthesia. Vitamin K antagonist treatment was not interrupted, while non-vitamin K anticoagulants were skipped on the morning of the procedure. A decapolar catheter (e.g., Dynamic XT™, Boston Scientific, Marlborough, MA, USA) was used to cannulate the coronary sinus. After single or double transseptal punctures under fluoroscopic guidance, intravenous unfractionated heparin boluses were administered, in order to maintain an activated clotting time of > 300 s. Intracardiac echocardiography probe was not used in any procedure. The basket mapping catheter (Orion™, Boston Scientific, Marlborough, MA, USA) and the ablation catheter (Stablepoint™ catheter, Boston Scientific, Marlborough, MA, USA) were then inserted. A standard, non-steerable sheath was used. The Orion™ catheter was used in combination with the Rhythmia™ HDx mapping system (Rhythmia™, Boston Scientific, Marlborough, MA, USA) to create a 3-dimensional electro-anatomical voltage and activation map of the left atrium. Mapping and ablation were primarily carried out in sinus rhythm; in patients in AF, electrical cardioversion was attempted in order to restore sinus rhythm, at the beginning of the procedure and before re-mapping. Point-by-point RF delivery was performed in such a way as to create contiguous ablation spots encircling the PVs. CF settings were at the individual operator’s discretion, within the range of 5 to 40 g. Ablation was guided by the magnitude and time course of impedance drop during RF delivery. RF applications were targeted to a minimum LI drop of 15 Ω within 15 s and were stopped when a maximum cutoff LI drop of ≥ 40 Ω was observed. We aimed to reach an LI drop of 20–30 Ω, on the basis of previous experimental data [8]. Radiofrequency energy was applied in the power controlled mode (45–50 W) with a temperature limit of 43°C. The irrigation rate was 30 ml/min during applications and 2 ml/min during mapping. A normal saline solution (NaCl 0.9%) was used. The recommended maximum distance between adjacent ablation spots (center-to-center) was ≤ 6 mm. The ablation points were marked automatically with 6-mm diameter, numbered AutoTags™. The starting impedance, initial CF, LI drop during RF, and average force applied were recorded. The endpoint of ablation was PV isolation, as assessed on the basis of entry and exit block by means of the 64-pole Orion™ catheter placed sequentially in each of the PVs. In the absence of first-pass PV isolation (i.e., no isolation upon completion of the encirclement of ipsilateral veins), PV isolation was accomplished by means of additional RF applications at the investigator’s discretion.

### Local impedance

**Fig. 1:**
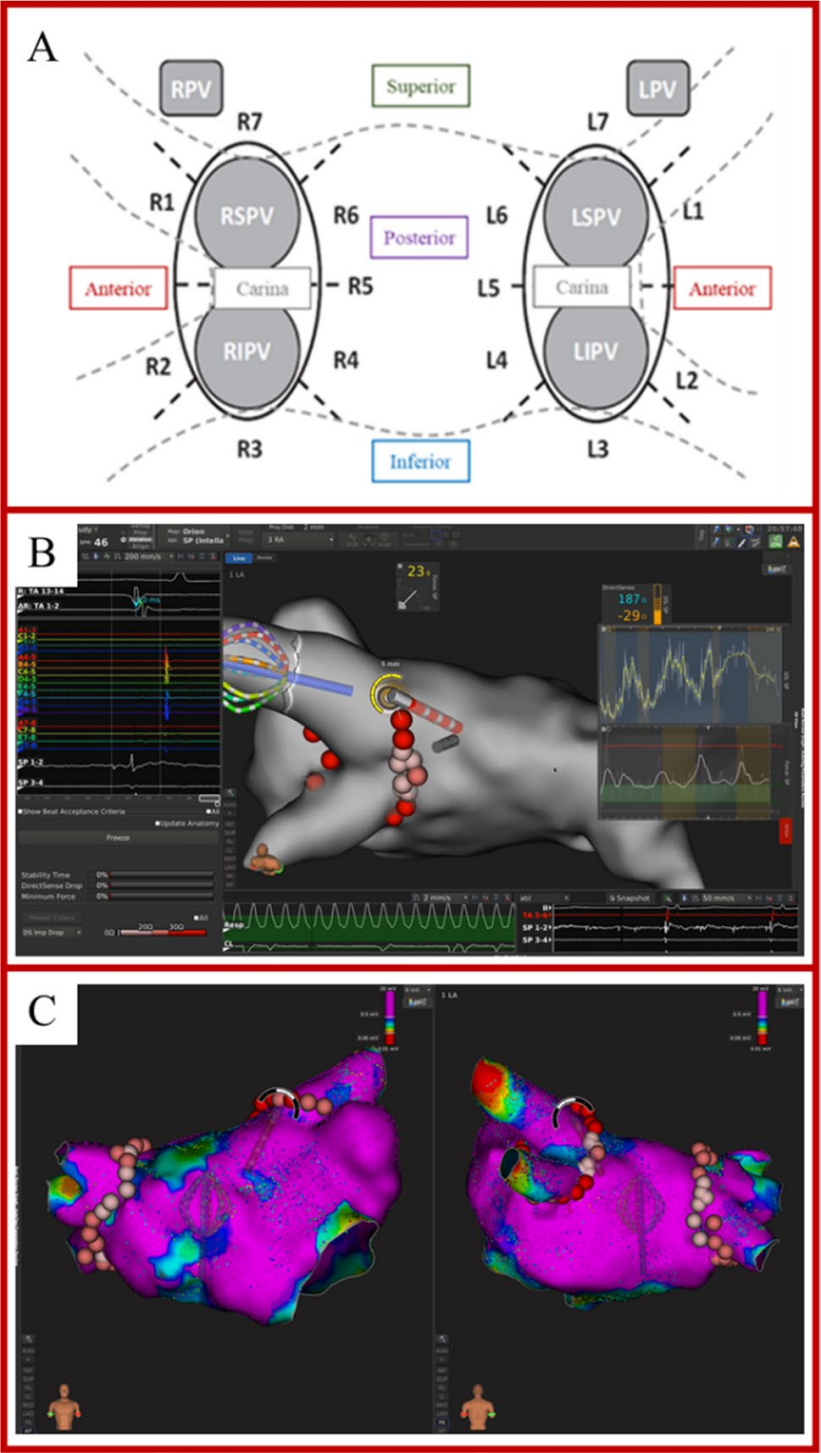
**A** Identification of 7 ablation sites around the right (RPV) and left (LPV) pairs of pulmonary veins. Anterior superior: R1, L1. Anterior inferior: R2, L2. Inferior: R3, L3. Posterior inferior: R4, L4. Carina: R5, L5. Posterior superior: R6, L6. Superior: R7, L7. LIPV = left inferior pulmonary vein; LSPV = left superior pulmonary vein; RIPV = right inferior pulmonary vein; RSPV = right superior pulmonary vein. **B** Example of visualization of CF and DirectSense™ tool on the Rhythmia™ mapping system during ablation. **C** Point-by-point RF delivery created contiguous ablation spots encircling the PVs. The maximal inter-lesion distance between two neighboring lesions was set ≤ 6 mm and was automatically measured through the Autotag™ software. CF settings were at the individual operator’s discretion, within the range of 5 to 40 g

A 3-electrode method with separate circuits for field creation and measurement was used to measure LI. As previously described, non-stimulatory alternating current was delivered between the tip electrode and the proximal ring; voltage was passively measured between the tip electrode and the distal ring [9]. As the catheter used does not have mini-electrodes, the resulting voltage was measured from the catheter tip. Impedance was calculated by dividing the voltage by the stimulatory current. To measure the baseline reference impedance of the blood pool, once the reference map had been completed, the ablation catheter was positioned in the blood pool for 10 s, and the value was calculated when no EGM recordings from the ablation catheter were present. Baseline tissue impedance and impedance drop for each ablation lesion were measured. To analyze the impedance information, the isolation line around each pair of PVs was divided into seven distinct sections (Fig. 1A) in accordance with the literature [10]. Videos of the ablation procedures were exported from the mapping system, to display the procedure in real time. RF current applications were then retrospectively analyzed.

### Contact force

The ablation catheter used in the current study has the ability to measure both real-time LI calculated from a local electric field generated at the tip of the catheter and CF. The force applied to the tip electrodes transferred to inductive sensors via a spring. The signal change measured by the inductive sensors is then converted to a 3-dimensional force vector by means of known spring dynamics. The target CF was 5–40 g, at the operator’s discretion. We collected the following data on each first-pass ablation point: power, minimum CF, maximum CF, mean CF, duration of application, baseline LI, and LI drop. In addition, the CF range during the applications was calculated by subtracting the minimum CF from the maximum CF of the ablation point. All numbered AutoTag™ points were exported from the system for off-line analysis. An example of visualization of CF values and the DirectSense™ tool on the Rhythmia™ mapping system during ablation is depicted in Fig. 1B and C.

### Follow-up

Complications were reported on the case report form and collected during follow-up. After ablation, anticoagulation and antiarrhythmic drugs therapy were continued. At 3 months, anticoagulation was continued according to the stroke risk, whereas antiarrhythmic drugs were continued at the discretion of the treating physician. Clinical evaluation and ECG were performed at 1, 3, 6, and 12 months. Holter ECG was performed at 3, 6, and 12 months post-ablation or in the case of symptoms. For the purpose of this study, data were collected during the index procedure and during an ambulatory visit 30 days after the procedure.

### Statistical analysis

Descriptive statistics are reported as means ± SD for normally distributed continuous variables or medians with 25th to 75th percentiles in the case of skewed distribution. Normality of distribution was tested by means of the non-parametric Kolmogorov-Smirnov test. Differences between mean data were compared by means of a *t*-test for Gaussian variables, and the *F*-test was used to check the hypothesis of equality of variance. The Mann-Whitney non-parametric test was used to compare non-Gaussian variables. Differences in proportions were compared by applying *χ*^2^ analysis or Fisher’s exact test, as appropriate. Linear regression analysis was performed to determine relationships between LI drop, CF, and RF delivery time (DT). A *p* value < 0.05 was considered significant for all tests. All statistical analyses were performed with STATISTICA software, version 7.1 (StatSoft, Inc., Tulsa, OK).

## Results

### Study population and procedural parameters

The demographic and procedural data of the 45 consecutive *de novo* PV isolation patients are reported in Table 1. Almost two-thirds of the patients suffered from paroxysmal AF (*n* = 26, 58%), whereas 19 (42%) had a history of persistent AF. The mean procedure duration and fluoroscopy times were 107.4 ± 39 min and 11.1 ± 8 min, respectively. A total of 3196 RF applications were delivered, with a mean number of 64 ± 31 ablation spots during a mean RF delivery time of 8.7 ± 4 s, without any steam popping.

**Table 1:** Baseline characteristics and procedural parameters

Parameter	*n* = 45
Age, years	61.6 ± 9
Male Gender, *n* (%)	28 (62.2)
• Paroxysmal AF, *n* (%)	• 26 (57.8)
• Persistent AF, *n* (%)	• 19 (42.2)
History of atrial flutter/atrial tachycardia, *n* (%)	6 (13.3)
LVEF, %	55.1±8
Cardiomyopathy, *n* (%)	15 (33.3)
Hypertension, *n* (%)	26 (57.8)
Coronary artery disease, *n* (%)	5 (11.1)
History of heart failure, *n* (%)	3 (6.7)
COPD, *n* (%)	2 (4.4)
CKD, *n* (%)	1 (2.2)
ACE-ARB, *n* (%)	14 (31.1)
Beta-blockers, *n* (%)	28 (62.2)
Statin, *n* (%)	7 (15.6)
Diuretics, *n* (%)	5 (11.1)
Antiarrhythmics, *n* (%)	33 (73.3)
Procedure duration, min	107.4±39
Fluoroscopy time, min	11.1±8
RFC applications, *n*	64±31
RFC duration time, sec	8.7±4
Mean Power, W	47.2±3
Complications during the procedure, n (%)	0 (0)
Minor complications	3 (6.6)
• Mild pericardial effusion • Groin hematomas	• 1 • 2

*AF* atrial fibrillation, *PVI* pulmonary vein isolation, *LVEF* left ventricular ejection fraction, *RFC* radiofrequency catheter, *COPD* chronic obstructive pulmonary disease, *CKD* chronic kidney disease, *ACE* angiotensin-converting enzyme, *ARB* angiotensin receptor blocker

### Local tissue impedance values

High-quality data were available on 2895 (91%) RF applications performed around PVs. The baseline LI was 157.9 ± 17 Ω prior to ablation and 136.9 ± 14 Ω after ablation (*p* < 0.0001, absolute LI drop of 23.0 ± 7 Ω) with an LI drop rate of 3.5 ± 2 Ω/s. The mean blood-pool impedance was 152.7 ± 10 Ω (*p* < 0.0001 vs baseline LI). The magnitude of the impedance drop was predicted by the baseline LI (correlation coefficient *r* = 0.61, 95% confidence interval (CI): 0.59–0.63, *p* < 0.0001). Regarding AF type, no difference in baseline LI was found (158 ± 17 Ω for paroxysmal AF vs 157.9 ± 17 Ω 157.7 ± 17 Ω for persistent AF, *p* = 0.3878), whereas LI drops were larger in paroxysmal AF cases (23.3 ± 7 Ω) than in persistent AF cases (22.7 ± 7 Ω, *p* = 0.0097). On considering the underlying rhythm, no differences were found in terms of either baseline LI or LI drop (baseline LI: 157.2 ± 17 Ω for sinus rhythm vs 158.4 ± 17 Ω for AF, *p* = 0.0518; LI drop: 22.8 ± 7 Ω for sinus rhythm vs 22.9 ± 7 Ω for AF, *p* = 0.8606).

### Correlation between local impedance and key procedural parameters

**Fig. 2:**
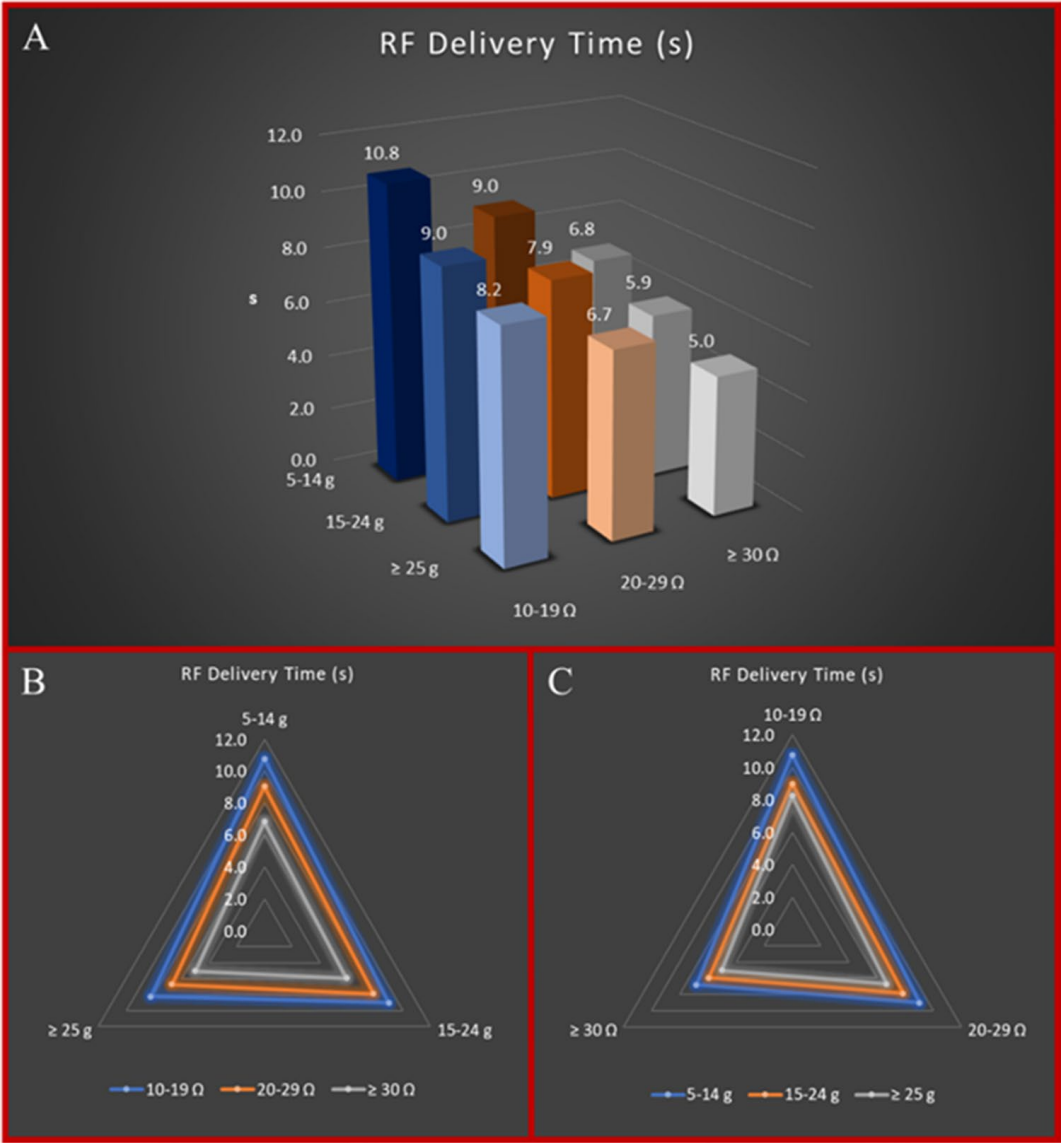
**A** Multidimensional relationship between RF delivery time, CF values, and LI drop values. **B** Radar plot showing the relationship between RF delivery time and CF values according to different degrees of LI drop. This Kiviat chart displays multivariate data (RF delivery time) with values represented on axes starting from the same point. The apexes of the Kiviat charts represent different CF intervals (5–14 g, 15–24 g, and ≥ 25 g), whereas the lines represent different degrees of LI drop (blue line for 10–19 Ω LI drop values, orange line for 20–29 Ω LI drop values and grey line for LI drop values ≥ 30 Ω). RF delivery time is represented according to CF and LI drop intervals. **C** Radar plot showing the relationship between RF delivery time and LI drop values according to different degrees of CF. This Kiviat chart displays multivariate data (RF delivery time) with values represented on axes starting from the same point. The apexes of the Kiviat charts represent different LI drop intervals (10–19 Ω, 20–29 Ω, and ≥ 30 Ω), whereas the lines represent different degrees of CF (blue line for 5–14 g CF values, orange line for 15–24 g CF values, and grey line for CF values ≥ 25 g). RF delivery time is represented according to CF and LI drop intervals

**Fig. 3:**
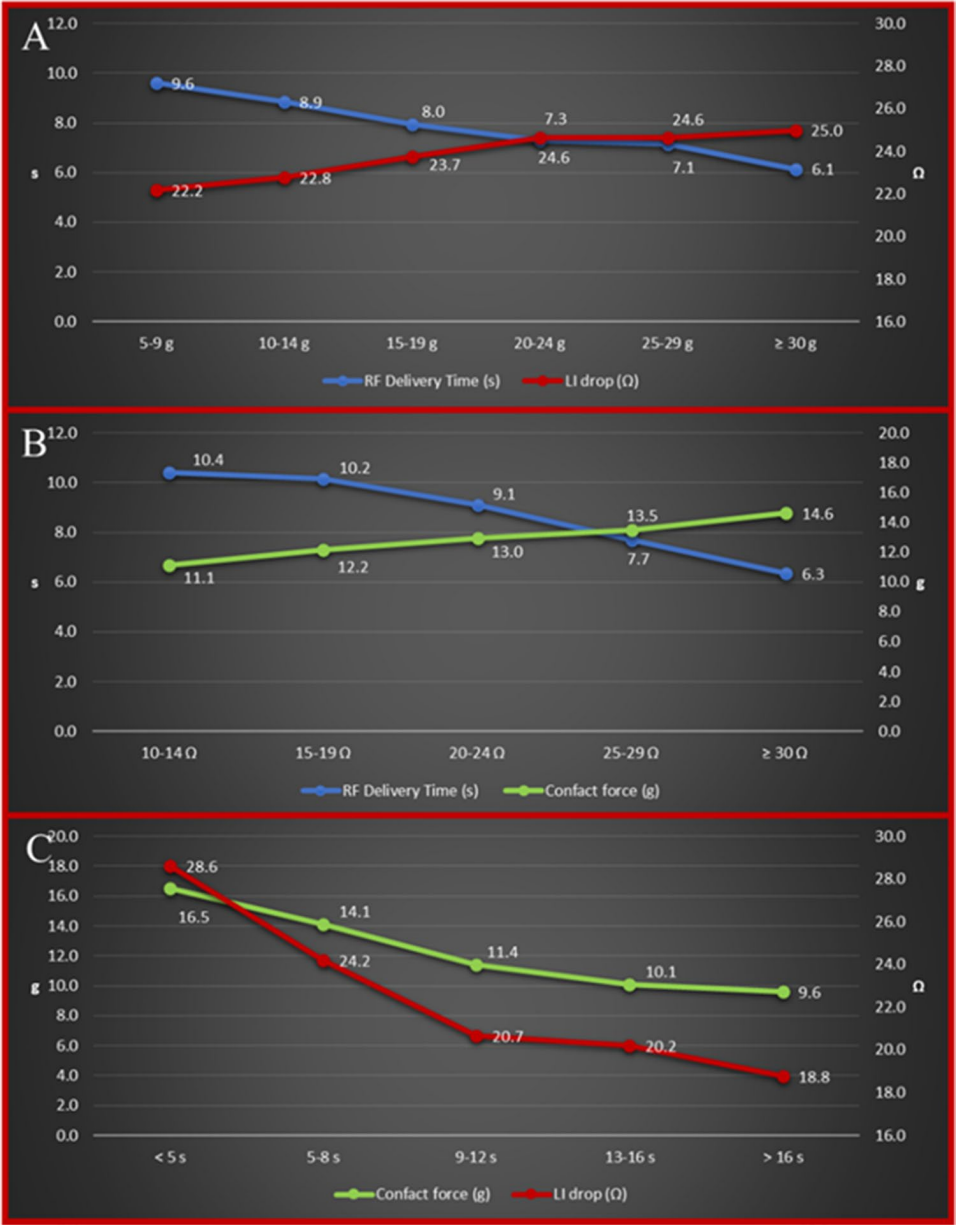
Details of relationships among the three key parameters: RF delivery time and LI drop according to different levels of CF (**A**); RF delivery time and CF according to different degrees of LI drop (**B**); and CF and LI drop according to different values of RF delivery time (**C**)

The mean RF delivery time was 8.7 ± 4 s, and the mean CF was 13.0 ± 8 g. On assessing the various key ablation parameters, the magnitude of LI drop proved to be weakly correlated with CF (*r* = 0.13, 95%CI: 0.09 to 0.16, *p* < 0.0001), whereas both CF and LI drop inversely correlated with DT (*r* = −0.26, 95%CI: −0.29 to −0.22, *p* < 0.0001 for CF; *r* = −0.36, 95%CI: −0.39 to −0.33, *p* < 0.0001 for LI). Fig. 2A to C show the resulting mean DT stratified by CF values and LI drop values. For each 10 g of CF, LI drops markedly increased from 22.4 ± 7 Ω to 24.0 ± 8 Ω at 5 to 25 g CF intervals (5–14 g of CF vs 15–24 g of CF, *p* < 0.0001), whereas it showed a smooth transition above 25 g (24.8 ± 7 Ω at ≥ 25 g CF intervals, *p* = 0.0606 vs 15–24 g of CF) (Supplementary Fig. 1). There was a correlation between shorter DT and larger drop in LI: 27.2 ± 8 Ω at 0–5 s of DT interval vs 22.8 ± 7 Ω at 6–10 s of DT interval vs 19.7 ± 6 Ω at > 10 s of DT interval (all comparisons *p* < 0.0001). Details of the relationships among the three parameters are reported in Fig. 3.

### Characterization of pulmonary vein location sites

**Fig. 4:**
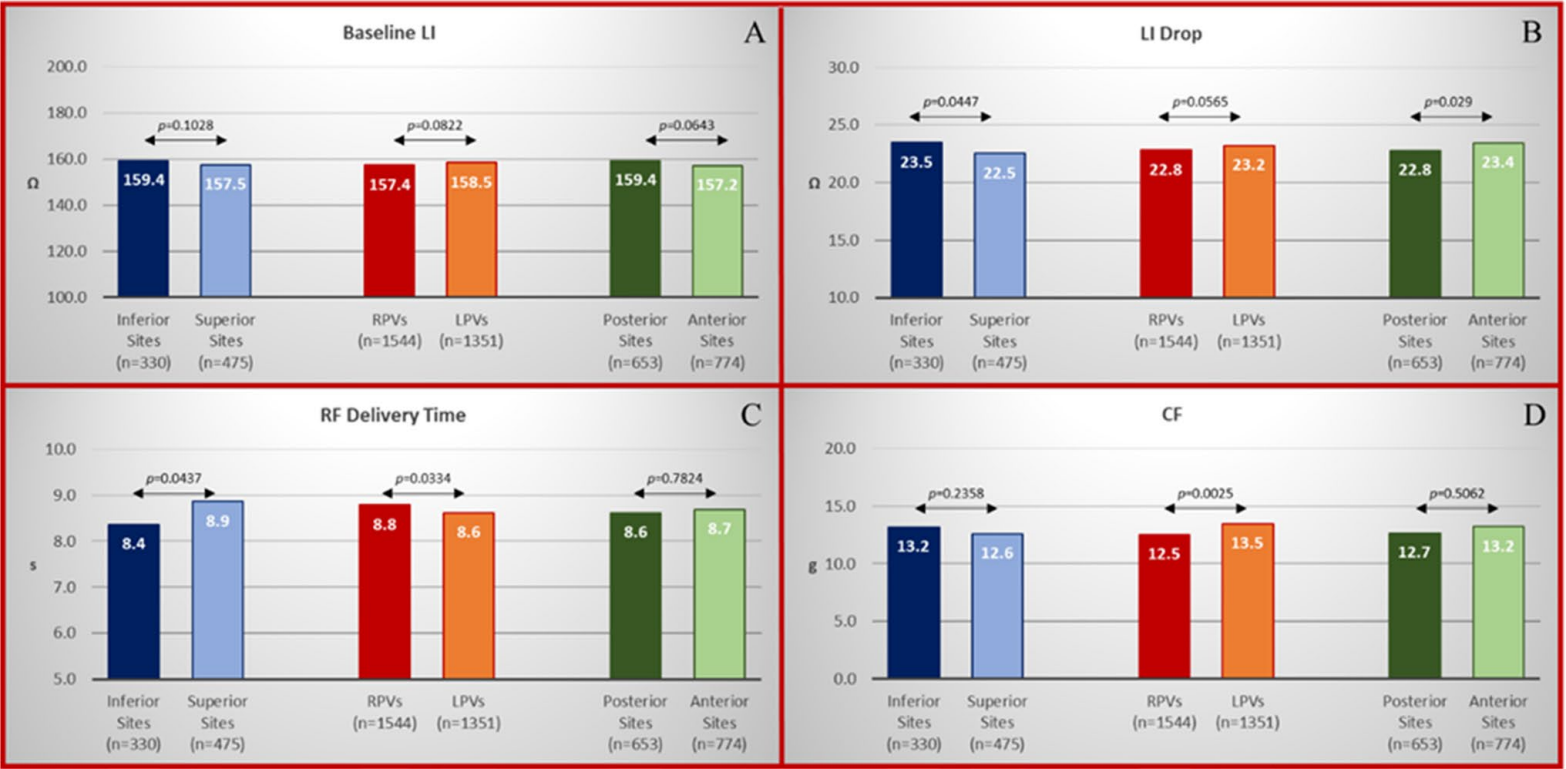
Details of the distribution of baseline LI (**A**), LI drop (**B**), RF delivery time (**C**), and CF values (**D**) according to location sites: anterior sites vs posterior sites, LPV sites vs RPV sites and inferior vs superior sites

**Fig. 5:**
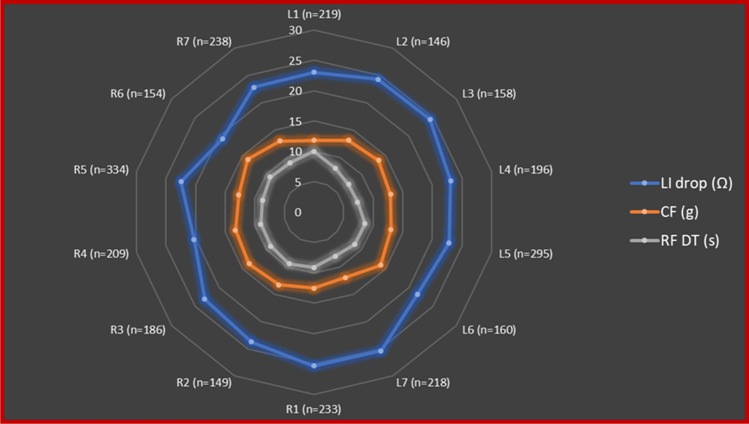
Details of the distribution of RF applications, CF, ablated tissue impedance values, and RF delivery times according to location sites. This Kiviat chart displays multivariate data with values represented on axes starting from the same point. Each apex of the Kiviat charts represents a location site according with seven distinct sections of right (R) and left (L) pairs of PVs (anterior superior R1, L1; anterior inferior R2, L2; inferior R3, L3; posterior inferior R4, L4; carina R5, L5; posterior superior R6, L6; superior R7, L7). Blue, orange, and grey dots represent the mean LI drop values, CF values, and RF delivery time values according to location sites

Of the 2895 RF applications, 1544 (53.3%) were sited on the RPVs and 1351 (46.7%) on the LPVs. Baseline impedance was homogenous across the various location sites (158.5 ± 17 Ω at LPVs vs 157.4 ± 17 Ω at RPVs, *p* = 0.0822; 157.2 ± 17 Ω at anterior sites vs 159.4 ± 18 Ω at posterior sites, *p* = 0.0643; and 159.4 ± 17Ω at inferior sites vs 157.5 ± 15Ω at superior sites, *p* = 0.1028). LI drop was higher at anterior sites (23.4 ± 7 Ω vs 22.8 ± 8 Ω at posterior sites, *p* = 0.029) and at inferior sites (23.5 ± 7 Ω vs 22.5 ± 7 Ω at superior sites, *p* = 0.0447), whereas it was similar between RPVs and LPVs (22.8 ± 7 Ω in RPV pairs vs 23.2 ± 7 Ω at LPVs, *p* = 0.0565) (Fig. 4A, B). RF delivery time was longer at superior sites (8.9 ± 4 s vs 8.4 ± 4 s at inferior sites, *p* = 0.0437) and in RPV pairs (8.8 ± 4 s vs 8.6 ± 4 s in LPV pairs, *p* = 0.0334), whereas no differences were found between posterior and anterior sites (8.6 ± 4 s at posterior sites vs 8.7 ± 4 s at anterior sites, *p* = 0.7824) (Fig. 4C). CF values were higher in LPV pairs (13.5 ± 8 g vs 12.5 ± 7 g in RPV pairs, *p* = 0.0025), whereas no differences were found between superior and inferior sites (13.2 ± 8 g vs 12.6 ± 8 g, *p* = 0.2358) or between posterior and anterior sites (12.7 ± 7 g vs 13.2 ± 8 g, *p* = 0.5062) (Fig. 4D). Details of the distribution of RF applications, CF values, LI drops, and RF delivery times, according to location sites, are reported in Fig. 5 and Supplementary Table 1. Details of baseline and ablated tissue impedance values are reported in Supplementary Fig. 2 and Supplementary Table 1.

### First pass isolation and acute outcome

No steam pops or major complications, including atrio-esophageal fistula or tamponade, were reported during or after the procedures. In our series, a total 169 PVs (94%) were isolated at first pass ablation, resulting in 40 (89%) patients who had a first pass isolation, whereas 11 residual gaps in 5 (11%) patients were observed after initial encirclement and required additional RF applications. LI drop values were larger and CF values were higher where first pass isolation was achieved (LI drop 23.1 ± 7 Ω at successful sites vs 16.8 ± 3 Ω at unsuccessful sites, *p* < 0.0001; CF 13 ± 8 g at successful sites vs 10.2 ± 6 g at unsuccessful sites, *p* = 0.0207, respectively). At the end of the procedures, all PVs had been successfully isolated in all study patients. Minor complications were reported in 3 patients (6.6%) after the procedure: one pericarditis with mild pericardial effusion, and groin hematomas in 2 patients. Conservative treatment and medical therapy were effective in all cases, without prolongation of hospital stay.

## Discussion

### Main findings

In this single-arm prospective study, we performed AF catheter ablation by means of a novel ablation catheter with integrated CF- and LI-sensing capabilities. The ablation strategy, which was guided by LI information, had a 100% acute procedural effectiveness rate, without causing any steam pops or major complications. CF significantly impacted on effective lesion formation during RF PV isolation. The use of higher than 25 g contact between the catheter and the tissue proved to have less impact on LI drop. The inverse correlation of both CF and LI drop with RF DT indicates that a significant reduction in RF time can be achieved at 45–50 W power in a point-by-point workflow when LI guidance is combined with CF. These points reflect the value of LI plus CF in discerning both mechanical contact and electrical coupling, thereby enabling safe and effective lesions to be created.

### Ablation guided by local impedance and contact force

The use of highly localized impedance measurements to provide insight into tissue characteristics and their real-time evaluation seems to be helpful in order to precisely assess the electrical contact of the catheter and tip stability and to serve as a viable real-time indicator of tissue characteristics and durability of the lesions created [5–7, 11]. Two commercially available catheters capable of recording LI are currently available. The IntellaNav MiFi OI catheter (Boston Scientific) generates LI measurements through mini-electrodes on the tip of the ablation catheter, the maximum value being reported within a three-dimensional mapping environment (Rhythmia; Boston Scientific). A recently released StablePoint catheter (Boston Scientific) incorporates CF-sensing capability in addition to LI [8]. The ablation strategy for PV isolation guided by LI technology has proved safe and effective, resulting in a very low rate of AF recurrence over 1-year follow-up [7]. However, as the dedicated ablation catheter (IntellaNAV Mifi OI, Boston Scientific) used in these studies was not able to collect data on CF sensing, it was not possible to compare CF and impedance measurements.

It is well recognized that, when RF energy is applied, CF is one of the variables, in addition to catheter stability, power output, temperature, and duration of RF output, that impacts on lesion size and transmurality [4]. CF-guided RF catheter ablation has been associated with a significantly greater AF/atrial tachycardia-free survival benefit than non-CF-guided ablation in patients with paroxysmal AF rather than persistent AF. In addition, the CF-guided ablation strategy also reduced procedure time, fluoroscopy time, and RF time, though it had no distinct effect on the alleviation of procedure-related complications [12]. Adding CF sensing to the LI-sensing technology has the potential to further increase the efficiency of LI-guided catheter ablation. Indeed, we found that CF significantly impacted on effective lesion formation during RF PV isolation. However, the benefit of higher than 25 g contact between the catheter and the tissue had less impact on the increase in LI drop. Our findings may have relevant implications in the clinical setting: [1] good catheter-tissue contact improves the drop in LI and shortens the time needed to achieve it; [2] the lack of benefit of a CF value of above 25 g might avoid excessive catheter pressure and potential complications. Similar data have already been reported with other CF-sensing technologies [10, 13]; [3] the CF value may help to differentiate the LI value of the blood pool from that of diseased tissue. Indeed, both the blood pool and diseased tissue display lower LI values than healthy tissue [5, 14]. Of note, the magnitude of the mean LI drop observed in our study (23.0 ± 7 Ω) was significantly higher than that reported with previous LI technology (IntellaNAV Mifi OI, Boston Scientific) by other authors: Segreti et al. [5], 14 ± 8 Ω; Das et al. [6], 19.8 ± 11.1 Ω, and Solimene et al. [7], 13 ± 8 Ω. To date, only one pilot study, which used the StablePoint™ ablation catheter [15], showed that a local impedance drop > 21.8 Ω on the anterior wall and > 18.3 Ω on the posterior wall significantly increased the probability of creating a successful lesion. The CF-LI catheter does not have microelectrodes; instead, its distal tip serves as the return pole of the LI circuit. The larger electrical field created gives rise to CF-LI values that are typically 40–50% greater than those measured by the non-CF-LI catheter [16]. Further studies will therefore be required in order to determine the magnitude of LI drop that predicts acute PV segment conduction block.

### Right power, right duration

Winkle et al. first showed that AF ablations can be performed at 45–50 W for short durations with very low complication rates. High-power, short-duration ablations have the potential to shorten procedural and total RF times and to create more localized and durable lesions [17]. In addition, high-power short-duration RF ablation has proved able to significantly shorten procedure time, fluoroscopy time, left atrial dwell time, and RF ablation time in comparison with a conventional approach, with no difference in safety outcomes between the two groups [18]. When high-power short-duration ablation is performed, several parameters can indicate that a lesion has been formed and that no further ablation is needed, thereby avoiding ablation for longer than needed to selectively destroy the target tissue. These parameters include the following: monitoring the loss of pacing capture during RF delivery, observing a drop in impedance, and following such metrics of lesion formation as the lesion size index or the ablation index [19–21]. Our findings showed an inverse correlation of both CF and LI drop with DT, together with a significant reduction in RF time at 45–50 W power in a point-by-point workflow. This reflects the value of LI plus CF in discerning both mechanical contact and electrical coupling, thereby enabling safe and effective lesions to be created.

### Limitations

This investigation focused on the effect of each single RF application, and no data on medium- and long-term clinical outcomes were available. Impedance drop was used to assess lesion formation; however, it is only a surrogate and could be affected by several factors. The LI values that we used were empirically chosen. However, they were based on our previous experience in clinical practice, in which they had allowed us to achieve considerable clinical success. Further studies are required to identify the best workflow and targeted parameters also for achieving long-term success. The effect of using a steerable sheath during ablation may need further investigation. Lastly, esophageal temperature monitoring was not performed. However, in our preliminary experience, applying this procedural workflow, no steam pops or major complications, including atrio-esophageal fistula or tamponade, occurred during or after the procedures.

## Conclusions

CF significantly impacts on effective lesion formation during RF PVI. The benefit of higher than 25 g contact between the catheter and the tissue appears to have less impact on LI drop.

## Supplementary Information

ESM 1 (DOCX 276 kb)

## Data Availability

The data underlying this article will be shared on reasonable request to the corresponding author.
