# Supplementary material for: When local impedance meets contact force: preliminary experience from the CHARISMA registry
Source: J Interv Card Electrophysiol. 2022 Mar 24;63(3):749–58. doi: 10.1007/s10840-022-01163-7 (PMC9151535; doi:10.1007/s10840-022-01163-7)

**Supplementary material**

**Supplementary Table 1.** Details of the distribution of RF applications, CF, baseline and ablated tissue impedance values according to location sites

| **Location Site** | | | **n (%)** | **Baseline LI, Ω** | **LI drop, Ω** | **CF, g** | **RF application time, sec** |
| --- | --- | --- | --- | --- | --- | --- | --- |
| RPV | Anterior superior | R1 | 233 (8.0) | 161.1±18 | 25.4±7 | 12.5±7 | 9.2±4 |
|  | Anterior inferior | R2 | 149 (5.1) | 153.9±18 | 23.7±8 | 13.3±8 | 9.5±4 |
|  | Inferior | R3 | 186 (6.4) | 159.0±21 | 23.0±7 | 13.7±9 | 9.1±4 |
|  | Posterior inferior | R4 | 209 (7.2) | 157.0±16 | 20.3±7 | 13.3±8 | 9.0±4 |
|  | Carina | R5 | 334 (11.5) | 156.1±18 | 22.4±8 | 12.7±7 | 8.7±4 |
|  | Posterior superior | R6 | 154 (5.3) | 155.3±13 | 19.3±6 | 13.9±10 | 9.2±4 |
|  | Superior | R7 | 238 (8.2) | 159.0±16 | 22.8±7 | 13.0±7 | 9.0±4 |
| LPV | Anterior superior | L1 | 219 (7.6) | 154.3±16 | 23.0±8 | 11.8±7 | 10.0±5 |
|  | Anterior inferior | L2 | 146 (5.0) | 157.7±16 | 24.3±7 | 13.1±8 | 8.1±3 |
|  | Inferior | L3 | 158 (5.5) | 157.9±15 | 24.5±8 | 13.6±10 | 7.3±4 |
|  | Posterior inferior | L4 | 196 (6.8) | 158.3±15 | 23.1±6 | 13.0±7 | 7.4±4 |
|  | Carina | L5 | 295 (10.2) | 156.8±17 | 22.8±8 | 13.0±8 | 8.6±4 |
|  | Posterior superior | L6 | 160 (5.5) | 158.7±16 | 21.8±7 | 14.1±8 | 8.6±4 |
|  | Superior | L7 | 218 (7.5) | 164.9±18 | 25.4±7 | 12.0±6 | 8.1±4 |

CF=contact force; LI=local impedance; RF=radiofrequency; RPV=right pulmonary vein; LPV=left pulmonary vein.

**Supplementary figure 1.** Details of LI drop values according to different values of CF. Although there is a significant difference dichotomizing CF values below and above 25 grams (22.8±7Ω at < 25g CF intervals vs 24.8±7Ω at ≥25g CF intervals, p<0.0001), LI drops markedly increased from 5 to 25g CF intervals, whereas it showed a smooth transition above 25g. No differences were found comparing 20-24g CF interval to both 25-29g and ≥25g CF intervals. Polynomial trendline is displayed in red.


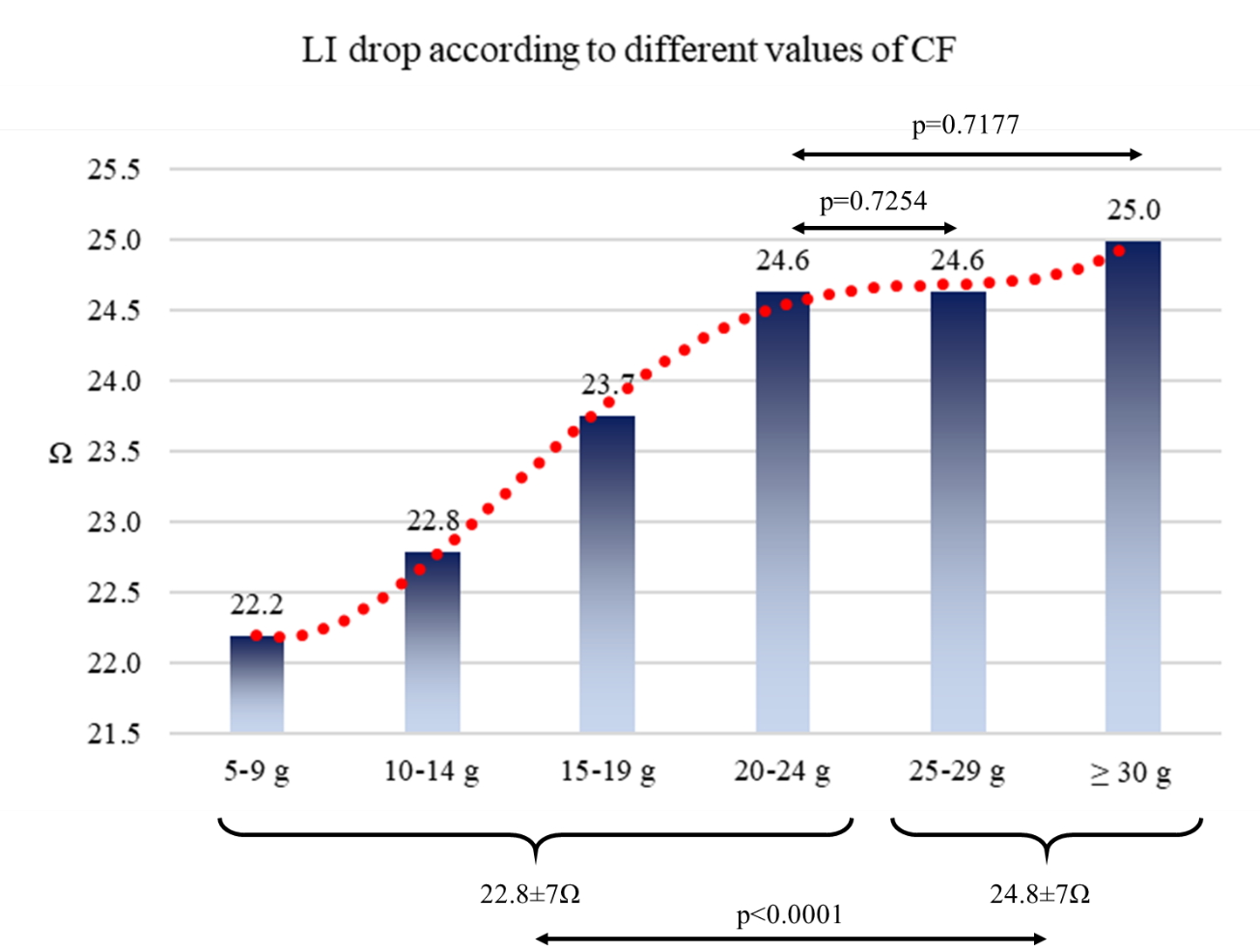


**Supplementary figure 2.** Details of the distribution of baseline and ablated tissue impedance values according to location sites with seven distinct sections of right (R) and left (L) pairs of PVs. Anterior superior: R1, L1; Anterior inferior: R2, L2; Inferior: R3, L3; Posterior inferior: R4, L4; Carina: R5, L5; Posterior superior: R6, L6; Superior: R7, L7. The mean baseline LI and ablated tissue impedance values at sites of left pair of PVs are reported in red tones, at sites of right pair of PVs are reported in blue tones. In green tones are reported the mean values at all sites. Dark color denotes baseline LI, light color denotes ablated tissue impedance.


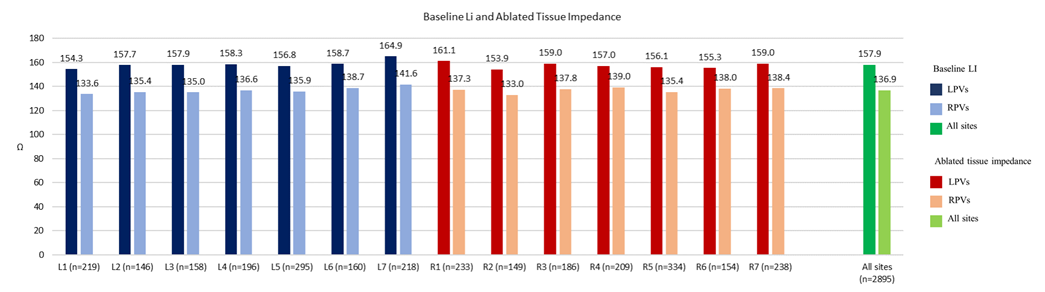

Supplement: Supplementary file 1 — (DOCX 276 kb) [file 10840_2022_1163_MOESM1_ESM.docx]
